# FTIR-based spectroscopic analysis in the identification of clinically aggressive prostate cancer

**DOI:** 10.1038/sj.bjc.6604753

**Published:** 2008-11-04

**Authors:** M J Baker, E Gazi, M D Brown, J H Shanks, P Gardner, N W Clarke

**Affiliations:** 1 Manchester Interdisciplinary Biocentre, Centre for Instrumentation and Analytical Science, School of Chemical Engineering and Analytical Science, The University of Manchester, Manchester, M1 7DN, UK; 2 Genito Urinary Cancer Research Group, School of Cancer and Imaging Sciences Paterson Institute for Cancer Research, University of Manchester, Wilmslow Road, Manchester, M20 4BX, UK; 3 Department of Histopathology, Christie Hospital NHS Foundation Trust, Manchester, M20 4BX, UK; 4 Department of Urology, Christie Hospital NHS Foundation Trust, Manchester, M20 4BX, UK; 5 Department of Urology, Salford Royal NHS Foundation Trust, Salford, M6 8HD, UK

**Keywords:** FTIR microspectroscopy, prostate cancer, molecular diagnosis, Gleason grade, PC-DFA, infrared spectroscopy

## Abstract

Fourier transform infrared (FTIR) spectroscopy is a vibrational spectroscopic technique that uses infrared radiation to vibrate molecular bonds within the sample that absorbs it. As different samples contain different molecular bonds or different configurations of molecular bonds, FTIR allows us to obtain chemical information on molecules within the sample. Fourier transform infrared microspectroscopy in conjunction with a principal component-discriminant function analysis (PC-DFA) algorithm was applied to the grading of prostate cancer (CaP) tissue specimens. The PC-DFA algorithm is used alongside the established diagnostic measures of Gleason grading and the tumour/node/metastasis system. Principal component-discriminant function analysis improved the sensitivity and specificity of a three-band Gleason score criterion diagnosis previously reported by attaining an overall sensitivity of 92.3% and specificity of 99.4%. For the first time, we present the use of a two-band criterion showing an association of FTIR-based spectral characteristics with clinically aggressive behaviour in CaP manifest as local and/or distal spread. This paper shows the potential for the use of spectroscopic analysis for the evaluation of the biopotential of CaP in an accurate and reproducible manner.

Prostate cancer (CaP) is the most common cancer and second most common cause of cancer-related deaths of men in the United Kingdom, with 10 164 deaths in 2003 (Cancer Research UK, 2006). The diagnosis of CaP and in particular, the prediction of biopotential in individual patients can be very problematic. Biopotential refers to the ability of the disease to progress to severe cancer. This has led to an inappropriate treatment and pathological upstaging in some cases and an overtreatment of biologically indolent disease in others (Parker *et al*, 2006).

Confirmation of initial diagnosis involves a biopsy and use of the Gleason grading system for histological analysis. This technique, which is the mainstay for diagnosis and prediction of tumour biopotential, utilises the Gleason grading system (Gleason, 1977) based on tumour morphology, identifying a complex continuum of glandular architecture enabling the grouping of cancer types into five visually distinct grades. This technique, although widely used, has well-recognised flaws. In particular, there are many theoretically low-grade/‘low-biopotential’ tumours, which show evidence of progression and in addition, there is considerable inter- and intra-observer error in the assignation of the final tumour grade. In a study of 390 patients, identical grades were assigned to only 29.2% of tumours (Latouf and Saad, 2002), and a UK-based study using 81 CaP slides found that on first readings the consensus score was only 78% (Melia *et al*, 2006).

The limitation with these diagnostic modalities has led to an interest in the development of spectroscopic analytical techniques for the diagnosis of cancer. Infrared (IR) spectroscopy is a non-destructive method for the analysis of tissues, fluids and cells (Sahu and Mordechai, 2005). Infrared radiation causes vibration of the bonds of the molecules within the sample that absorbs it. The wavelength of the IR radiation absorbed depends on the atoms involved in the bond and the strength of any intermolecular interactions. Therefore, each molecule can have a different spectrum; in essence, the IR spectrum is a fingerprint of the sample. Infrared spectra of biomolecules allow the measurement of complex molecular vibrational modes that contain valuable information on changes occurring due to diseases such as cancer. Infrared spectroscopy is a quick, cost-effective, simple-to-operate, reagent-free technique that requires simple sample preparation.

Fourier transform infrared (FTIR) spectroscopy coupled with advanced computational methods has been used to detect many different forms of cancer from tissue biopsies. These include benign and malignant prostate (Gazi *et al*, 2003, 2004, 2006), colon (Lasch *et al*, 2002) and cervical (Wood *et al*, 2004) tissues, all of which have been evaluated using this technique and with a high rate of diagnostic accuracy. In relation to grading of CaP, the sensitivities and specificities reported earlier have been as high as 78 and 89%, respectively, using a Fourier transform infrared-linear discriminant analysis model to predict Gleason scores less than 7 (GS<7), equal to 7 (GS=7) and greater than 7 (GS>7) (Gazi *et al*, 2006).

This paper discusses the use of FTIR microspectroscopy combined with principal component-discriminant function analysis (PC-DFA) to evaluate formalin-fixed archival CaP tissue based upon a three-band and a two-band Gleason score criteria. The aim of this paper is to determine the possible biochemical changes associated with the progression of CaP and to observe whether there are characteristic spectroscopic changes between prostate confined and locally invasive tumours.

## Materials and methods

### Primary tissue preparation and sampling for FTIR

With full ethical committee approval, Trent MREC 01/4/061, tissue was collected from 39 consenting patients undergoing transurethral resection of the prostate for bladder outflow obstruction. Forty CaP tissue biopsy specimens from 39 men were obtained as paraffin-embedded blocks (Genito-Urinary Cancer Research Group, Paterson Institute for Cancer Research) from patients with CaP. The tissue was fixed in 4% formalin for 24 h, then placed onto a Thermoshandon Excelsior processor that passes 20, 90 and four 100% ethanol aliquots through the tissue for an hour each, followed by three changes of xylene for an hour each at a temperature of 40°C, followed by three changes of paraplast for an hour each at 62°C. Then the tissue was embedded in a mould of molten wax and cooled on an ice plate for an hour before storage.

Serial sections were collected at 10 *μ*m thickness from each specimen, one of which was mounted onto a BaF_2_ plate (Linkham Scientific Ltd, Tadworth, UK) with the adjacent section mounted onto a glass slide and stained with haematoxylin and eosin (H&E). An experienced histopathologist with a subspecialist interest in genitourinary pathology, including participation in the UK national prostate histopathology external quality assurance scheme, assigned Gleason scores to areas of malignancy identified within the H&E sections. The complementary sections of the same cancer region were mounted onto BaF_2_ plates and washed on an orbital mixer with citroclear for 6 min to remove the paraffin and then acetone at 4°C for a further 6 min before being air-dried for 1 h under ambient conditions. The anatomical features identified from the H&E section were used as landmarks to position the IR beam upon the malignant lesions of the unstained adjacent section.

### FTIR microspectroscopy

Fourier transform infrared spectra of Gleason-graded primary prostate tissues were collected in transmission mode using a Nicole Magna system 550 spectrometre equipped with a liquid nitrogen-cooled MCT/A detector and a KBr beam splitter. The spectrometer is attached to a microscope equipped with a video camera to view optical images ( × 150 magnification) of the sampling area and a programmable computerised *x*–*y* stage. An aperture size of 60 × 60 *μ*m was used to collect malignant epithelial cell spectra. Fourier transform infrared spectra represent an average of 512 scans in the mid-IR wavenumber range 750–4000 cm^−1^ with a spectral resolution of 4 cm^−1^. Background scans were obtained from a region outside the sample field and ratioed against the sample spectrum. In total, 395 FTIR spectra were obtained from the 40 CaP tissue biopsies from 39 patients with at least five spectra recorded from each patient. The total number of spectra acquired from each patient was dependent on the size of the tumour lesion within the tissue section as well as on the positive identification of tumour glands in the unstained tissue section.

### Data processing

**Figure 1:**
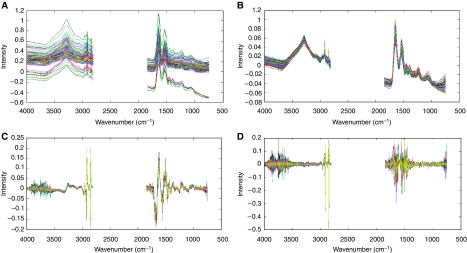
Raw spectral data (**A**) and the effect of three pre-processing steps above on an example subset of the FTIR data with the variable CO_2_ region removed. (**B**) Vector normalisation (model A), (**C**) vector normalisation combined with first derivatisation (model B) and (**D**) vector normalisation combined with second derivatisation (model C).

Data processing was carried out immediately after spectral acquisition using OMNIC v.5.1a software. OMNIC software was used to convert the FTIR absorbance spectra into first derivatives (Savitzky–Golay algorithm, nine smoothing points) and second derivatives. The spectral region used was 750.1859–3999.706 cm^−1^ with the variable CO_2_ region from 1847.501 to 2809.22 cm^−1^ removed. This resulted in 1188 spectral data points for analysis. The spectra were then vector normalised in Matlab™, to correct for baseline shifts (scattering). Three different pre-processing procedures were used on raw spectra and assessed for optimum discrimination; these steps were (1) vector normalisation (model A), (2) vector normalisation combined with first derivatisation (model B) and (3) vector normalisation combined with second derivatisation (model C). Figure 1 shows raw spectral data and the effect of three pre-processing steps above on an example subset of the FTIR data with the variable CO_2_ region removed. Matlab coupled with an in-house written software was used to perform PC-DFA. Principal component-discriminant function analysis uses principal component analysis to reduce the dimensionality of the data before DFA. Discriminant function analysis then discriminates between groups on the basis of the resulting PCs and the *a priori* knowledge of the group memberships that are fed into the DFA algorithm (Baker *et al*, 2008). Maximising the inter-group variance and minimising the intra-group variance achieve this. The maximum number of discriminant functions available is the number of groups minus one. The optimum number of PCs was determined through an iterative process of chemometric model generation and validation. Discriminant function analysis is a supervised technique and the model is supplied with information about group membership, so any result produced by the model needs to be tested. This testing was carried out by retaining one-fifth of the total filtered pre-processed spectra, randomly selected as an independent test set, and then supplying the spectra to the model as a test set and observing where the model places the spectra on a graphical output. Error ellipses with 95 and 90% confidence are added to the discriminant function plots. This was achieved using error_ellipse.m written by AJ Johnson and obtained from Matlab central file exchange. Covariance matrices were calculated from the discriminant function analysis score matrix for each grouping, where the centroid was defined as the mean of the discriminant function analysis score matrix for each grouping.

## Results

### Correlation of FTIR PC-DFA spectral signature with Gleason score

The FTIR PC-DFA diagnostic model was derived from 40 CaP tissue biopsies. The 40 biopsies correspond to 395 spectra (90 spectra from 10 biopsies for GS<7, 118 spectra from 11 biopsies for GS=7 and 187 spectra from 19 biopsies for GS>7). Spectra were divided into a training set consisting of 315 spectra (72 spectra from GS<7, 94 spectra from GS=7 and 149 spectra from GS>7) and a randomly chosen test set consisting of a fifth of the total data set of 80 spectra (18 spectra from GS<7, 24 spectra from GS=7 and 38 spectra from GS>7). The CaP diagnostic model was built on the training set data and it was at this stage that spectroscopic similarities and differences between spectra collected from different Gleason scores were determined by the PC-DFA algorithm, in a supervised manner. The test set was then used to interrogate the model classification of the different spectra.

The Gleason score is the sum of the predominant and highest Gleason grades observed within a biopsy. When the biopsy has only one Gleason grade, this grade is doubled to produce the resulting Gleason score (Gleason, 1977). A three-band Gleason score criterion was used that divided specimens into groups corresponding to GS<7, GS=7 and GS>7. This method is a more clinically orientated scale in which Gleason scores <7 are less aggressive, Gleason=7 are of intermediate biopotential and Gleason scores >7 are the most likely to progress. This grouping has been shown earlier to be characterised by specific FTIR spectral characteristics (Gazi *et al*, 2006). Table 1 shows the sensitivity and specificity observed for each of the bands in the criterion at 95% and stringent 90% confidence limits for models obtained using three different data analysis pre-processing steps. These models are (A) spectra vector normalised, (B) spectra vector normalised followed by first derivative and (C) spectra vector normalised followed by second derivative. Sensitivity refers to the proportion of people with disease who have a positive test result and specificity refers to the proportion of people without the disease who have a negative test result.

**Figure 2:**
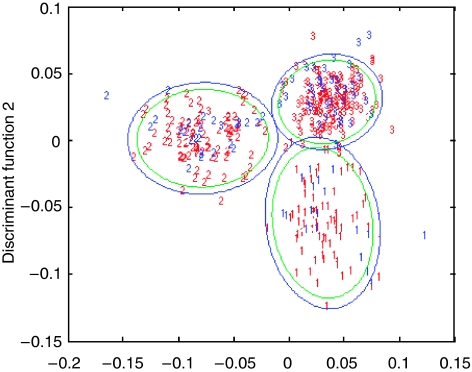
Discriminant function plot showing separation of model B (first derivative vector normalised) based upon the training set (red digits) and test set (blue digits) with 95% (blue ellipse) and 90% (green ellipse) confidence limits, where 1=Gleason score less than 7, 2=Gleason score equal to 7 and 3=Gleason score greater than 7. DFA – DF1 *vs* DF2.

Model B (vector normalised combined with first derivative) achieves the greatest overall sensitivity of 92.3% for the models at the 95% confidence limit. The overall sensitivity is the average of the three measurements attained when discriminating the three groups (GS<7, GS=7 and GS>7). Figure 2 shows the discriminant function plot for model B showing separation based upon the training set (red digits) and test set (blue digits) with 95% (blue ellipse) and 90% (green ellipse) confidence limits, where 1=GS<7, 2=GS=7 and 3=GS>7.

**Figure 3:**
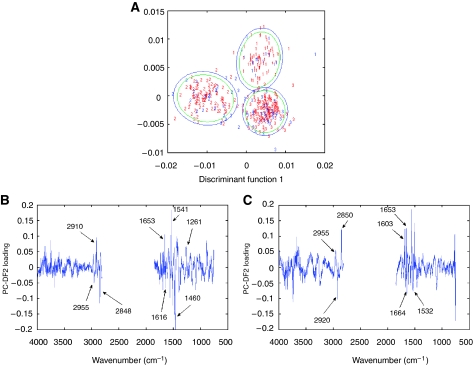
(**A**) Discriminant function plot showing separation of model A (vector-normalised model) based upon the training set (red digits) and test set (blue digits) with 95% (blue ellipse) and 90% (green ellipse) confidence limits, where 1=Gleason score less than 7, 2=Gleason score equal to 7 and 3=Gleason score greater than 7. (**B**) Loading plots for discriminant function 1 and (**C**) discriminant function 2. DFA – DF1 *vs* DF2.

Discerning the peaks responsible for discrimination from factor loadings based upon a first derivative spectral model is particularly difficult as the peak in the raw spectrum becomes a point on the baseline in the first derivative. The use of the first derivative in pre-processing spectra removes the additive baseline shift and enhances the chemical information available for discrimination. However, as model A achieves the highest overall specificity of 99.4% at the 95% confidence limit from spectra that have only been vector normalised (to remove baseline shifts), model A can be used to indicate which peaks are responsible for discrimination. Figure 3A shows the discriminant function plot for model B based upon training and test set data with confidence ellipses as in Figure 2.Figure 3B and C shows the loading plots for discriminant functions 1 and 2 from which we obtain the spectral peaks responsible for discrimination in model A.

The axes of the discriminant function plot have positive and negative directions (Figure 3). The peaks in the positive direction of the *y* axis of the DF1 loading plots correspond to the spectral peaks that the model is using to discriminate GS<7 and GS>7 from GS=7, and the peaks in the negative direction are used to discriminate GS=7 from GS<7 and GS>7. The peaks in the positive direction of discriminant function 2 correspond to the spectral peaks used to discriminate GS<7 from GS>7, and peaks in the negative direction are used to discriminate GS>7 from GS<7. Table 2A shows the major spectral peaks responsible for the Gleason score discrimination and proposed biomolecular assignments for discriminant function 1 and Table 2B for discriminant function 2.

### Correlation of FTIR PC-DFA spectral signature with clinical stage

The clinical stage is taken from the tumour/node/metastases classification system. For the purpose of this experiment, we are concerned with information from the primary tumour, investigating T1 and T2 tumours (confined to the prostate), T3 tumours (breaching the prostate capsule or invading the seminal vesicle) and T4 tumours (extending beyond the prostate and seminal vesicle to invade local pelvic structures). N stages are classified as N0, Nx (nodal status unknown) or N+ (nodal status positive) and M stage as M0 or M1. Definitive lymph node staging was not available in T1 and T2 patients: these were designated N0. For the same reasons, most low-stage and -grade tumours were designated Mx.

Clinical staging information was available for 33 out of 39 patients. This corresponds to 347 spectra (191 spectra from 18 biopsies for T1 and T2 and 156 spectra from 15 biopsies for T3 and T4). The spectra were split into a training set consisting of 276 (152 spectra from T1 and T2 and 124 spectra from T3 and T4) spectra and a randomly chosen test set consisting of a fifth of the total data set of 71 spectra (39 spectra from T1 and T2 and 32 spectra from T3 and T4). No weighting criteria were applied to the spectra, so each spectrum is of equal importance. Different number of spectra from different groups in the model does not bias the model as we are discriminating upon differences between groups and grouping on similarities within groups.

In our study, a two-band criterion was used that divided specimens into either stage T1 and T2 (confined to the prostate) or T3 and T4 (extension outside the prostate). The sensitivity and specificity calculations were based upon spectral assignment to these criteria. If the spectrum was predicted to lie within the correct group then this was counted as a positive prediction. Table 3 shows the sensitivity and specificity observed for each of the bands in the criterion at 95% and more stringent 90% confidence limits for (A) model A – spectra vector normalised, (B) model B – spectra vector normalised followed by first derivative and (C) model C – spectra vector normalised followed by second derivative.

**Figure 4:**
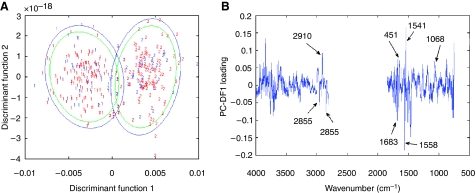
(**A**) Discriminant function plot showing separation based on model A (vector-normalised model) upon the training set (red digits) and test set (blue digits) with 95% (blue ellipse) and 90% (green ellipse) confidence limits, where 1=T1 and T2 and 2=T3 and T4, and (**B**) loading plots for discriminant function 1. DFA – DF1 *vs* DF2.

The best-performing model is model A, which attained overall sensitivity and specificity of 91.2 and 93.0%. This model used 62 PCs and one discriminant function. Figure 4A shows the discriminant function plot based upon the training set (red digits) and test set (blue digits) with 95% (blue ellipse) and 90% (green ellipse) confidence limits, where 1=T1 and T2, and 2=T3 and T4. Figure 4B shows the loading plots for discriminant function 1, from which it is possible to obtain the peaks responsible for discrimination. As the model has only two groups, only one discriminant function is sufficient for the separation.

The peaks in the positive direction of discriminant function 1 are used to discriminate T3 and T4 from T1 and T2, and the peaks in the negative direction are used to discriminate T1 and T2 from T3 and T4. Table 4 shows the major spectral peaks and possible biomolecular assignments from discriminant function 1 that are used to discriminate the two-band criteria.

## Discussion

### Correlation of FTIR PC-DFA spectral signature with Gleason score

Gleason grading of CaP biopsies is subject to both intra- and inter-observer variability, which reduces any prognostic value arising from the grading system (Latouf and Saad, 2002). Fourier transform infrared vibrational spectroscopy determines the chemical profile of a specimen by measuring the entire chemical spectrum by means of absorbance of specific wavenumbers of IR light, which relate to specific chemical bonds. These specific absorbencies are due to bond vibrations, which correlate with individual chemical groups. Interrogation and cross correlation of these enable the acquisition of information detailing the chemical constituents of local area of tissue within a biopsy (Diem *et al*, 2004).

To reduce the variability in Gleason grades between specimens, we graded our specimens using a single histopathologist to assign Gleason scores to CaP tissue sections. Latouf and Saad (2002) reported that there was only 29.2% agreement in the assignment of Gleason score to biopsy and radical prostatectomy samples when assessment was made by multiple pathologists. However, the same study showed that when samples were assessed by a single pathologist, the agreement rose to 48.7%. They concluded that assessment by the same pathologist reduces the discrepancy in the Gleason grading of prostate biopsy.

Although Gleason grading is a useful clinical method, there are clear difficulties with inter-observer variation. Additional diagnostic modalities may provide an important adjunct to diagnosis and, in particular, to the specific identification of tumours with aggressive biopotential. We have shown in earlier studies (Gazi *et al*, 2006) that chemometric analysis using vibrational spectroscopy is a viable tool for assessing paraffin-embedded formalin-fixed CaP biopsies. This study has developed the technique still further, resulting in a significant improvement in the overall sensitivity and specificity. This was 73 and 86.3% in the earlier work (Gazi *et al*, 2006) as compared with 92.3 and 98.9% achieved in this study.

One of the limitations with this study and the study by Gazi *et al* (2006) is that the Gleason grading system was used as the reference standard for the development of the diagnostic algorithms. This methodology is inevitably flawed, as it will tend to incorporate problems inherent in the Gleason system into the new system. However, it was felt to be important to investigate this novel area in this manner to establish proof of principle. This goal has now been achieved, with the data currently presented showing clearly that FTIR-based methods can identify and discriminate between CaPs of different types. A study utilising archival tissue from several different tissue banks, increasing the number of cases, would provide a greater test for the discrimination. Future study will now concentrate upon the development of a spectral grading scale in anticipation that this approach may yield alternative and potentially complementary methods for the assessment of CaP.

One of the promising features of these results was the ability to identify a difference in the tissue characteristics of locally confined and locally invasive tumours. The spectral differences between the Gleason scores (Table 2 and Figure 3) occur in the amide I (1600–1700 cm^−1^), amide II (1510–1580 cm^−1^) and amide III (1200–1400 cm^−1^) regions of the spectra. This suggests a difference in the protein content of the cancerous tissue as the cancer progresses to more aggressive states. Meurens *et al* (1996) show the amide I and amide II bands to be among the most significant in relation to breast carcinoma, Fabian *et al* (2006) utilise the amide II region to differentiate colorectal adenocarcinoma from non-cancerous tissue and Bamberry *et al* (2004) reported that the major spectral differences between the different cells in cervical cancer tissue predictably occur in the amide I region. The majority of the study in FTIR has focussed upon the ability to discriminate cancerous from non-cancerous tissue. Ductal carcinoma *in situ* has been differentiated from benign breast tissue, with 93% classified correctly (Fabian *et al*, 2006). Colorectal adenocarcinoma tissue (Lasch *et al*, 2002) has been investigated in this way with an overall classification accuracy of 95%. Both these studies used optimised artificial neural networks to process the spectral data. This study has developed spectroscopic analysis further by utilising a PC-DFA algorithm to identify the difference in tumour grade and to predict the absence of local invasion in CaP biopsies. The prediction of biopotential is of particular importance in CaP, where there is a pressing need to differentiate between the indolent and aggressive forms of the disease. The former represents the majority of cancers diagnosed through screening, whereas the latter is responsible for a large number of male cancer deaths (Albertsen *et al*, 2005; Parker *et al*, 2006). The results presented herein suggest that the differing biopotential of cancer may be determined by different protein structures corresponding to changes in the amide I peak in the FTIR spectra. The proposed spectral assignments for the positive direction of discriminant function 1 (the spectral peaks GS<7 and GS>7 from GS=7) have two spectral loading peaks that can be related to *α*-helical structures, whereas the peaks in the negative direction (the spectral peaks that discriminate GS=7 from GS<7 and GS>7) have amide I spectral peaks that can be related to *α*-helical structure and antiparallel *β*-sheets/aggregated strands.

The lipid region also aids the differentiation within discriminant function 1, with spectral loading peaks for both groups illuminating different peaks in the lipid region (Figure 3, Table 2). Differentiation based on lipid characteristics has already been proven useful in breast cancer in the differentiation of benign from malignant breast disease (Fabian *et al*, 2006). Discriminant function 2 is discriminating GS<7 and GS>7. The GS=7 centroid lies close to the zero point on the discriminant function 2 axis, showing that GS=7 has components that are present in both GS<7 and GS>7. As the GS=7 group is made from Gleason grades 3 and 4, the interrogation of only one of these grades in the CaP tissue will provide it with attributes of the GS<7 or GS>7 group. Using this discrimination to shed light upon the biochemicals responsible, we can stipulate that the GS<7 group uses spectral peaks that can be attributed to DNA/RNA and *α*-helical, unordered turns or antiparallel *β*-sheet protein structures and the GS>7 group uses *β*-sheet, aggregated strand, 3_10_ helical and turns protein structures. The major peaks in the loadings that could be used as a biomarker to distinguish aggressiveness of cancer occur in discriminant function 2. Peaks at 1653 and 1683 cm^−1^, which are attributable to amide I peaks (Table 2B), are prominent in the loadings for GS<7 and peaks 2920 and 1577 cm^−1^, attributable to amide II and C-H stretch asymmetric (Table 2B), are prominent in the loadings for GS>7.

### Correlation of FTIR PC-DFA spectral signature with clinical stage

To our knowledge, this is the first time that FTIR spectroscopy has been shown to correlate with the local biopotential of CaP. The importance of the identification of tumour aggression has already been documented, but the data from this study also suggest that it may be possible to facilitate the identification of those patients with T3 and T4 disease from those with disease that is truly localised to the prostate. This is a potentially important finding that may be of particular utility in treatment planning, where pathological upstaging of disease previously thought to be of lower stage is a common problem. The FTIR PC-DFA discrimination shows that there is a valid biochemical difference between the two groups of T stages. This may be useful not only for interrogation of CaP tissue, but also in the field of biochemical marker development.

The spectral loading peaks responsible for this important discrimination between the two-band T stage criteria (Table 3 and Figure 4) again are based in the amide and lipid regions, with T1 and T2 using DNA/RNA and *α*-helical structures and T3 and T4 using C-O and C-C stretches, C-O-H and C-O-C deformations of carbohydrates and aggregated strands, and *α*-helical and antiparallel *β*-sheet protein structures. The major peaks in the loadings that could be used as a biomarker to indicate local aggressiveness are at 1558 cm^−1^, attributable to amide II (for less aggressive locally) (Table 4), and 1541 cm^−1^, attributable to amide II of *α*-helical structures (more aggressive locally) (Table 4). As with biochemical grading of CaP biopsies by FTIR, FTIR prediction of biopotential relies on detecting subtle changes in protein conformations relating to individual tumour behaviour.

In conclusion, this study reports the use of FTIR combined with PC-DFA to attain overall sensitivities and specificities of 92.3 and 98.9%, respectively, to predict Gleason scores <7, =7 and >7. These results represent a significant increase in accuracy when compared with the earlier reports. The results also show for the first time that a two-band criterion-based system identifies characteristics that differentiate between tumours that are clinically confined to the prostate from those that are clinically invasive. These findings have significant potential importance in the development of better techniques for the diagnosis, prognostication and treatment planning in CaP.

## Figures and Tables

**Table 1:** Sensitivities and specificities observed for less than 7, equal to 7 and greater than 7 Gleason score at 95 and 90% confidence limits and overall sensitivities and specificities for (i) model A (vector normalised), (ii) model B (vector normalised with first derivative) and (iii) model C (vector normalised with second derivative)

**Gleason score**	**GS<7**		**GS=7**		**GS>7**		**Overall**	
*(i)*								
Confidence limit	95%	90%	95%	90%	95%	90%	95%	90%
Sensitivity (%)	94.4	88.8	83.3	70.8	86.8	76.3	88.2	78.6
Specificity (%)	100	100	98.2	98.2	100	100	99.4	99.4
								
*(ii)*								
Confidence limit	95%	90%	95%	90%	95%	90%	95%	90%
Sensitivity (%)	88.9	83.3	95.8	91.7	92.1	89.5	92.3	88.2
Specificity (%)	98.4	100	98.2	98.2	100	100	98.9	99.4
								
*(iii)*								
Confidence limit	95%	90%	95%	90%	95%	90%	95%	90%
Sensitivity (%)	61.1	61.1	91.7	87.5	81.6	76.2	78.1	74.9
Specificity (%)	98.4	100	94.6	96.4	97.6	97.6	96.9	98

**Table 2:** Major spectral peaks responsible for the Gleason score discrimination and proposed biomolecular assignments for (A) discriminant function 1 and (B) for discriminant function 2

**Direction**	**Wavenumber (cm^−1^)**	**Proposed biomolecular assignment**
*(A)*		
+ve	1063	C-O stretch, deoxyribose/ribose, DNA, RNA
+ve	1261	Amide III (NH bend (55%), C-C stretch (19%), C-N stretch (15%), CO bend (11%))
+ve	1265	Amide III ((NH bend (55%), C-C stretch (19%), C-N stretch (15%), CO bend (11%)) or PO_2_^−^ stretch, RNA, DNA
+ve	1541	Amide II of *α*-helical structures (NH bend (43%), C-N stretch (29%), CO bend (15%), C-C stretch (9%), N-C stretch (8%))
+ve	1653	Amide I of *α*-helical structures or unordered (C-O stretch (76%), C-N stretch (14%), CCN deformation (10%))
+ve	2910	C-H stretch (asymmetric) of >CH_2_ in fatty acids, lipids, proteins
+ve	2920	C-H stretch (asymmetric) of >CH_2_ in fatty acids, lipids, proteins
+ve	2966	C-H stretch (asymmetric) of CH_3_ in fatty acids, lipids, proteins
−ve	1086	C-O, C-C stretches, C-O-H, C-O-C deformation of carbohydrates or PO_2_^−^ symmetric stretch of phosphodiester group in nucleic acids and phospholipids
−ve	1151	C-O, C-C stretch, C-O-H, C-O-C deformation of carbohydrates or C-OH stretch of serine, threonine, tyrosine in cell proteins
−ve	1460	CH_3_ antisymmetric bend
−ve	1473	CH_2_ scissoring
−ve	1616	Amide I of aggregated strand protein structures (C-O stretch (76%), C-N stretch (14%), CCN deformation (10%))
−ve	1650	Amide I of *α*-helical protein structures (C-O stretch (76%), C-N stretch (14%), CCN deformation (10%))
−ve	1686	Amide I of antiparallel *β*-sheet/aggregated strand protein structures (C-O stretch (76%), C-N stretch (14%), CCN deformation (10%))
−ve	2848	C-H symmetric stretch of >CH_2_ in fatty acids, lipids and proteins
−ve	2895	C-H stretch of C-H in methine groups
−ve	2955	C-H asymmetric stretch of -CH_3_ in fatty acids, lipids and proteins
		
*(B)*		
+ve	1065	C-O stretch, deoxyribose/ribose, DNA, RNA
+ve	1558	Amide II (NH bend (43%), C-N stretch (29%), CO bend (15%), C-C stretch (9%), N-C stretch (8%))
+ve	1653	Amide I of *α*-helical or unordered structures (C-O stretch (76%), C-N stretch (14%), CCN deformation (10%))
+ve	1683	Amide I of turns or antiparallel *β*-sheet structures (C-O stretch (76%), C-N stretch (14%), CCN deformation (10%))
+ve	2850	C-H symmetric stretch of -CH_2_ in fatty acids, lipids and proteins
+ve	2955	C-H antisymmetric stretch of -CH_3_ in fatty acids, lipids and proteins
−ve	1532	Amide II of *β*-sheets (NH bend (43%), C-N stretch (29%), CO bend (15%), C-C stretch (9%), N-C stretch (8%))
−ve	1577	Amide II (NH bend (43%), C-N stretch (29%), CO bend (15%), C-C stretch (9%), N-C stretch (8%))
−ve	1621	Amide I of aggregated strand structures (C-O stretch (76%), C-N stretch (14%), CCN deformation (10%))
−ve	1664	Amide I of turns 3_10_ helical structure (C-O stretch (76%), C-N stretch (14%), CCN deformation (10%))
−ve	1672	Amide I of turns structure (C-O stretch (76%), C-N stretch (14%), CCN deformation (10%))
−ve	2920	C-H stretch (asymmetric) of >CH_2_ in fatty acids, lipids, proteins

Spectral assignments taken from references Meurens *et al* (1996); Tamm and Tatulain (1997); Dovbeshko *et al* (2000); Naumann (2001); Cai and Ram Singh (2004); and Meade *et al* (2007).

**Table 3:** Sensitivities and specificities observed for T1 and T2 and T3 and T4 at 95 and 90% confidence limits and overall sensitivities and specificities for (A) vector normalised, (B) vector normalised with first derivative and (C) vector normalised with second derivative

**Clinical stage**	**T1 and T2**		**T3 and T4**		**Overall**	
*(A)*						
Confidence limit	95%	90%	95%	90%	95%	90%
Sensitivity (%)	94.9	92.3	87.5	84.4	91.2	88.4
Specificity (%)	91	91	94.9	97.4	93.0	94.2
						
*(B)*						
Confidence limit	95%	90%	95%	90%	95%	90%
Sensitivity (%)	92.3	89.7	84.4	78.1	88.4	83.9
Specificity (%)	87.5	90.6	97.4	97.4	92.5	94.0
						
*(C)*						
Confidence limit	95%	90%	95%	90%	95%	90%
Sensitivity (%)	92.3	89.7	84.4	81.3	88.4	85.5
Specificity (%)	84.4	84.4	92.3	92.3	88.4	88.4

**Table 4:** Major spectral peaks responsible for the 2-band discrimination and proposed biomolecular assignments for discriminant function 1

**Direction**	**Wavenumber (cm^−1^)**	**Proposed biomolecular assignment**
+ve	1068	C-O stretch, deoxyribose/ribose, DNA, RNA
+ve	1265	Amide III (NH bend (55%), C-C stretch (19%), C-N stretch (15%), CO bend (11%)) or PO_2_^−^ stretch, RNA, DNA
+ve	1415	C-N stretch, N-H, C-H deformation
+ve	1541	Amide II of *α*-helical structures (NH bend (43%), C-N stretch (29%), CO bend (15%), C-C stretch (9%), N-C stretch (8%))
+ve	1641	Amide I (C-O stretch (76%), C-N stretch (14%), CCN deformation (10%))
+ve	1645	Amide I (C-O stretch (76%), C-N stretch (14%), CCN deformation (10%))
+ve	1672	Amide I of turns structure (C-O stretch (76%), C-N stretch (14%), CCN deformation (10%))
+ve	2910	C-H stretch (asymmetric) of >CH_2_ in fatty acids, lipids, proteins
−ve	1244	Amide III (NH bend (55%), C-C stretch (19%), C-N stretch (15%), CO bend (11%))
−ve	1290	Amide III (NH bend (55%), C-C stretch (19%), C-N stretch (15%), CO bend (11%))
−ve	1364	Amide III (NH bend (55%), C-C stretch (19%), C-N stretch (15%), CO bend (11%))
−ve	1558	Amide II (NH bend (43%), C-N stretch (29%), CO bend (15%), C-C stretch (9%), N-C stretch (8%))
−ve	1655	Amide I of *α*-helical (C-O stretch (76%), C-N stretch (14%), CCN deformation (10%))
−ve	1683	Amide I of turns or antiparallel *β*-sheet structures (C-O stretch (76%), C-N stretch (14%), CCN deformation (10%))
−ve	1718	C-O stretch of carbonic acid
−ve	2850	C-H symmetric stretch of –CH_2_ in fatty acids, lipids and proteins
−ve	2918	C-H stretch (asymmetric) of >CH_2_ in fatty acids, lipids and proteins
−ve	2955	C-H antisymmetric stretch of –CH_3_ in fatty acids, lipids and proteins

Spectral assignments taken from references Meurens *et al* (1996); Tamm and Tatulain (1997); Dovbeshko *et al* (2000); Naumann (2001); Cai and Ram Singh (2004); and Meade *et al* (2007).

## References

[bib1] Albertsen PC, Hanley JA, Fine J (2005) 20-year outcomes following conservative management of clinically localized prostate cancer. JAMA 17: 2095–2101 10.1001/jama.293.17.2095 15870412

[bib2] Baker MJ, Brown MD, Gazi E, Clarke NW, Vickerman JC, Lockyer NP (2008) Discrimination of prostate cancer cells and non-malignant cells using secondary ion mass spectrometry. Analyst 133: 175–179 18227938 10.1039/b712853c

[bib3] Bamberry KR, Wood BR, Quinn MA, McNaughton D (2004) Fourier transform infrared imaging and unsupervised hierarchical clustering applied to cervical biopsies. Aust J Chem 57(12): 1139–1143

[bib4] Cai S, Ram Singh B (2004) A distinct utility of the Amide III infrared band for secondary structure estimation of aqueous protein solutions using partial least squares methods. Biochemistry 43: 2541–2549 14992591 10.1021/bi030149y

[bib5] Cancer Stats, Mortality-UK, Cancer Research UK (2006) http://info.cancerresearchuk.org/cancerstats/types/prostate/mortality/?a=5541 (26th January 2006)

[bib6] Diem M, Romeo M, Boydston-White S, Miljkovic M, Matthaus C (2004) A decade of vibrational micro-spectroscopy of human cells and tissue (1994–2004). Analyst 129: 880–885 15457314 10.1039/b408952a PMC2713762

[bib7] Dovbeshko GI, Gridina NY, Kruglova EB, Paschuk OP (2000) FTIR spectroscopy studies of nucleic acid damage. Talanta 53: 233–246 18968108 10.1016/s0039-9140(00)00462-8

[bib8] Fabian H, Thi NAN, Eiden M, Lasch P, Schmitt J, Naumann D (2006) Diagnosing benign and malignant lesions in breast tissue sections using IR-microspectroscopy. Biochim Biophys Acta 1758: 874–882 16814743 10.1016/j.bbamem.2006.05.015

[bib9] Gazi E, Dwyer J, Gardner P, Ghanbari-Siahkali A, Wade AP, Miyan J, Lockyer NP, Vickerman JC, Clarke NW, Shanks JH, Scott LJ, Hart CA, Brown M (2003) Applications of Fourier transform infrared microspectroscopy in studies of benign prostate and prostate cancer. A pilot study. J Pathol 201: 99–108 12950022 10.1002/path.1421

[bib10] Gazi E, Dwyer J, Lockyer N, Gardner P, Vickerman JC, Miyan J, Hart CA, Brown M, Shanks JH, Clarke N (2004) The combined application of FTIR microspectroscopy and ToF-SIMS imaging in the study of prostate cancer. Faraday Discuss 126: 41–59 14992399 10.1039/b304883g

[bib11] Gazi E, Baker M, Dwyer J, Lockyer NP, Gardner P, Shanks JH, Reeve RS, Hart CA, Clarke NW, Brown MD (2006) A correlation of FTIR spectra derived from cancer biopsies with Gleason grade and tumour stage. Eur Urol 50: 750–761 16632188 10.1016/j.eururo.2006.03.031

[bib12] Gleason DF (1977) In Urological Pathology—The Prostate, Tannenbaum M (ed), The Veteran's Administration Cooperative Urologic Research Group: histologic grading and clinical staging of prostatic carcinorma. Lee and Febiger: Philadelphia, p 171

[bib13] Lasch P, Haensch W, Lewis N, Kidder LH, Naumann D (2002) Characterization of colorectal adenocarcinoma sections by spatially resolved FT-IR microspectroscopy. Appl Spectrosc 48: 1–10

[bib14] Latouf JB, Saad F (2002) Gleason score on biopsy: is it reliable for predicting the final grade on pathology? BJU Int 90: 694–699 12410749 10.1046/j.1464-410x.2002.02990.x

[bib15] Meade AD, Lyng FM, Knief P, Byrne HJ (2007) Growth substrate induced functional changes elucidated by FTIR and Raman spectroscopy in *in-vitro*-cultured human keratinocytes. Anal Bioanal Chem 387B: 1717–1728 10.1007/s00216-006-0876-5 17102969

[bib16] Melia J, Moseley R, Ball RY, Griffths DF, Grigor K, Harnden P, Jarmulowicz M, McWilliam LJ, Montironi R, Waller M, Moss S, Parkinson MC (2006) A UK-based investigation of inter- and intra-observer reproducibility of Gleason grading of prostatic biopsies. Histopathology 48(6): 644–654 16681679 10.1111/j.1365-2559.2006.02393.x

[bib17] Meurens M, Wallon J, Tong J, Noel H, Haot J (1996) Breast cancer detection by Fourier transform infrared spectrometry. Vib Spectrosc 10: 341–346

[bib18] Naumann D (2001) FT-infrared and FT-Raman spectroscopy in biomedical research. Appl Spectrosc Rev 36(2&3): 239–298

[bib19] Parker C, Muston D, Melia J, Moss S, Dearnley D (2006) A model of the natural history of screen-detected prostate cancer and the effect of radical treatment on overall survival. Br J Cancer 94: 1361–1368 16641912 10.1038/sj.bjc.6603105 PMC2361275

[bib20] Sahu RK, Mordechai S (2005) Fourier transform infrared spectroscopy in cancer detection. Future Oncol 1(5): 635–647 16556041 10.2217/14796694.1.5.635

[bib21] Tamm LS, Tatulain SA (1997) Infrared spectroscopy of proteins and peptides in lipid bilayers. Q Rev Biophys 30(4): 365–429 9634652 10.1017/s0033583597003375

[bib22] Wood BR, Chiriboga L, Yee H, Quinn MA, McNaughton D, Diem M (2004) Fourier transform infrared (FTIR) spectral mapping of the cervical transformation zone, and dysplastic squamous epithelium. Gynaecol Oncol 93: 59–68 10.1016/j.ygyno.2003.12.028 PMC2732436 15047215

