# Supplementary material for: FTIR-based spectroscopic analysis in the identification of clinically aggressive prostate cancer
Source: Br J Cancer. 2008 Nov 4;99(11):1859–66. doi: 10.1038/sj.bjc.6604753 (PMC2600682; doi:10.1038/sj.bjc.6604753)
Supplement: Supplementary Information [file 6604753x1.doc]

**FTIR Based Spectroscopic Analysis in the Identification of Clinically Aggressive Prostate Cancer**

**Supplementary Information**

**10 Fold Cross Validation**

During a 10-fold cross validation the data is split into 10 sets of size n/10 where n equals the total number of spectra. The model is then ran at a range of retained principal components on nine of these datasets and testing on one dataset. This is repeated 10 times, changing the dataset used for testing each time and taking an average accuracy. Our accuracy is defined as the percentage correctly classified at a 95% confidence limit. The averaged accuracy for a range of retained principal components for the Gleason score model B (1^st^ derivative combined with vector normalisation) is shown below in Figure 1 and for the T stage model A (vector normalisation) in Figure 2, as these are the model which attained the highest overall sensitivities and specificities.

**Figure 1.** Average accuracy over a range of retained principal components of a 10-fold cross validation for the Gleason score model

**Figure 2.** Average accuracy over a range of retained principal components of a 10-fold cross validation for the T stage model

The 10-fold cross validation for the Gleason score model reveals that 59 principal components has the greatest average correctly classified and for the T stage model that 62 principal components has the greatest average correctly classified. These are the number of retained principal components utilised by the two models presented in the paper.
